# Impact of Spray-Dried Plasma on Intestinal Health and Broiler Performance

**DOI:** 10.3390/microorganisms7080219

**Published:** 2019-07-28

**Authors:** Joy M. Campbell, Joe D. Crenshaw, Ricardo González-Esquerra, Javier Polo

**Affiliations:** APC, Inc., 2425 SE Oak Tree Court, Ankeny, IA 50021, USA

**Keywords:** spray-dried plasma, poultry, broilers, antibiotics, performance

## Abstract

Spray-dried plasma (SDP) is a functional ingredient commonly utilized in swine production and calf milk replacers to improve performance, feed efficiency, and health. The improvements noted with SDP in animal production are more pronounced under commercial production conditions compared to cleaner research settings. Multiple modes of action of SDP have been proposed, including either directly influencing the immune inflammatory response locally or systemically, and/or through the indirect modification of beneficial microbial populations. Spray-dried plasma included at various dietary levels and duration of feeding in broilers has been evaluated in different production and challenging conditions with beneficial effects on broiler performance, as noted in other animals. The purpose of this review is to discuss research related to the modes of action of SDP on immunomodulation and improved intestinal health and specifically discuss research conducted utilizing SDP in feed for poultry. Collectively, the data available indicates that SDP improves early intestinal health and supports an efficient immune system response both locally at the intestine and systemically, thereby benefiting growth, feed efficiency, and survival of broilers in conventional commercial production and under challenging conditions such as disease or environmental stressors.

## 1. Introduction

Improving intestinal health and the efficiency of the immune system of animals impacts overall performance and health. In response to consumer perceptions, animal industry and public health concerns, the use of antibiotics is being restricted or eliminated for sub-therapeutic use in animal feed and new functional ingredients and antibiotic alternatives continue to be evaluated [1,2,3]. The identification of functional ingredients that may impact intestinal health and improve animal performance could be used as alternatives to help reduce reliance on sub-therapeutic antibiotics in animal feed and/or to advance current nutritional strategies.

Spray-dried plasma (SDP) is a dry functional ingredient obtained from blood collected and processed to preserve the functional characteristics of the proteins. Spray-dried plasma is a diverse mixture of many functional components [4,5] such as immunoglobulins, albumin, fibrinogen, lipids, growth factors, biologically active peptides, transferrin, enzymes, amino acids, and other factors that have biological activity systemically and/or within the intestine independent of their nutritional value.

Spray-dried plasma is extensively used in pig starter diets and calf milk replacers and provides a highly digestible protein in the formulation. Spray-dried plasma has consistently provided improvements in performance, feed efficiency, and animal survival. For a thorough review of these effects of SDP in pigs, the authors recommend reading the results summarized in several published review papers [4,6,7,8]. The beneficial effects of SDP are related to its mode of action of supporting an efficient immune system. There are multiple proposed modes of action of SDP, which include direct effects in the gastrointestinal tract [9] and systemic effects with impacts beyond the intestine such as on the respiratory [10] and reproductive systems [11].

The improvements noted with SDP in animal production are more pronounced under conventional commercial production conditions compared to cleaner research settings [12] and the authors concluded that the greater response to SDP in conventional production conditions may be related to the beneficial effects of SDP on intestinal health [12]. Likewise, similar results were reported in broilers [13] and turkeys [14]. Thus, understanding how SDP impacts local and systemic immune response can provide insight into its beneficial effects on intestinal health, function and poultry production.

The purpose of this review is to focus on the mode of action of SDP related to improving intestinal health and an efficient immune system and review research conducted utilizing SDP to improve overall performance and health in poultry.

## 2. Modes of Action of Spray-Dried Plasma

Intestinal inflammation results in a cascade of events such as edema, leukocyte infiltration, vasodilation, reduced nutrient absorption, increased epithelial permeability due to altered barrier function, and immune system activation. Intestinal inflammation may occur due to pathogen or environmental challenge, heat stress, antinutritional factors, and transportation stress. These types of stress activate the immune system resulting in pro-inflammatory cytokine release which triggers an inflammatory response that modulates changes in growth and metabolism [15,16,17]. Activation of the immune system requires increased nutrient use to maintain multiple immune system responders [18], thus making fewer nutrients available for productive functions such as growth and performance. Therefore, being able to improve the efficiency of the immune response by more rapidly restoring immune system homeostasis and maintaining the structural integrity of the intestinal barrier will more rapidly stabilize immune system activation and restore diverted nutrient use for productive functions.

Research conducted in mouse, rat, and pig models have evaluated the effects of SDP on intestinal inflammation and immune response but similar model studies using poultry-fed diets with SDP have not yet been published. In a challenge rat model, 8% SDP was formulated into the diets and orally fed for 14 days with challenge occurring on d 14 of the study by intraperitoneal injection of *Staphylococcus aureus* enterotoxin B (SEB) [19]. *Staphylococcus aureus* enterotoxin B is a known immune system activator resulting in a high percentage of activated T cells and intestinal inflammation [20]. After the challenge, SEB increased (*p* < 0.05) the water content of feces, which was prevented in rats consuming SDP, indicating that SDP can reduce the secretory effect of the SEB response. The SEB challenge also resulted in increased (*p* < 0.05) γδ-T lymphocytes and percentage of activated T helper cells in Peyer’s patches. These effects were partially prevented by the SDP diet, indicating that SDP can modulate the immune response by limiting immune activation that would impact the utilization of energy and nutrients. Nutrient absorption as indicated by measuring sodium glucose transporter 1 (SGLT1) was evaluated by Garriga et al. [21] in the SEB model. They reported an increase of 8–9% glucose absorption when rats were fed SDP and challenged with SEB compared to the SEB control group, indicating improvement of nutrient absorption when consuming SDP.

Peace et al. [22] reported that pigs fed a diet with 5% SDP for 14 days post-weaning had reduced intestinal inflammation as indicated by reduced (*p* < 0.05) intestinal tissue tumor necrosis factor α (TNFα) and lower (*p* < 0.05) fecal score along with improved intestinal barrier function as indicated by increased (*p* < 0.05) transepithelial electrical resistance, reduced (*p* < 0.05) short circuit current and reduced mucosal to serosal flux of mannitol and inulin. Thus, another mechanism of SDP on intestinal inflammation may be through improved barrier function and modulation of the mucosal immune response.

Utilizing the SEB rat model, Pérez-Bosque et al. [9] evaluated intestinal permeability and tight junction protein expression to assess both the structure and function of the intestine. The administration of SEB reduced (*p* < 0.05) the expression of tight junction proteins such as zonula occludens-1 (ZO-1) (10% reduction) and β-catenin (20% reduction) and increased the flux of both dextran and horse radish peroxidase (HRP) as tracers of mucosal permeability to toxins and food antigens. The increased permeability was due to a lower expression of tight junction proteins which was partially reduced with SDP. Feeding diets containing 8% SDP reduced the effects of SEB by reducing dextran and HRP flux across the intestinal epithelium. They concluded that SDP supplementation can in part reduce alterations of epithelium structure during intestinal inflammation, thereby improving intestinal mucosal barrier function, which may be reducing bacterial endotoxin translocation as indicated by the reduced flux of HRP which is similar in molecular weight to endotoxins.

Intestinal inflammation is mediated by cytokines and other pro- or anti-inflammatory mediators. Pérez-Bosque et al. [23,24] evaluated feeding 8% SDP in rats and mice on cytokine expression and mucosal lymphocyte activation and infiltration in the intestinal inflammation model and defensin expression. SEB increased (*p* < 0.05) the release of pro-inflammatory mediators (interferon γ (IFNγ), TNFα, Interleukin-6 (IL-6), and leukotriene B4 (LTB4)) in Peyer’s patches and intestinal mucosa which was prevented (*p* < 0.05) by feeding SDP. Feeding SDP also increased (*p* < 0.05) Interleukin 10 (IL-10) and mature transforming growth factor β (TGF-β) concentrations in intestinal mucosa. The administration of SEB also increased (*p* < 0.05) the expression of mucosal addressin cell adhesion molecule 1 (MAdCAM-1) and intercellular adhesion molecule 1 (ICAM-1) by 6-fold, with the effects being attenuated by the consumption of SDP. Furthermore, SEB increased nuclear factor kappa B (NF-κB) phosphorylation (*p* < 0.05), which was prevented (*p* < 0.05) by SDP and resulted in the reduction of leucocyte activation. SEB reduced (*p* < 0.05) the expression of β-defensin 1 and cryptdin 4 [25]. The supplementation of SDP prevented (*p* < 0.05) the reduction on expression of defensins caused by SEB resulting in restoration of defensin production and maintenance of the first line of defense in the intestinal mucosa. Thus, the SDP diet reduced the expression of TNFα, as well as, the phosphorylation of NF-κB and increased the expression of TGF-β and IL-10, suggesting a possible pathway of SDP immune modulation of restoring mucosal homeostasis and supporting anti-inflammatory effects.

In addition to the modulation of the intestinal immune response, dietary SDP has modes of action on systemic immunomodulation at other mucosal systems. There is extensive communication between the gut-associated lymphoid tissue (GALT) and other mucosal tissues such as nasal-associated lymphoid tissue (NALT) via the common mucosal immune system. Maijó et al. [10] evaluated feeding 8% SDP to mice challenged intranasally with lipopolysaccharide from *Escherichia coli* to induce acute lung inflammation. Bronchoalveolar lavage fluid (BALF) was collected to evaluate the systemic immune response affected by feeding SDP. Like effects noted in the intestine, the challenge from lipopolysaccharide (LPS) resulted in increased (*p* < 0.05) pro-inflammatory cytokines (TNFα, IL-6, Interleukin 1α (IL-1α)) and chemokines, while the feeding of SDP reduced the acute cytokine production by 20–80% and reduced (*p* < 0.05) chemokine ligands (CCL) CCL2, CCL3, and CCL4 production. With the adaptive immune response [26], LPS increased (*p* < 0.05) T helper lymphocytes in lung and blood that was reduced by feeding 8% SDP. LPS also reduced (*p* < 0.05) TGF-β concentrations in the lung with no effect on IL-10, while feeding SDP to mice increased (*p* < 0.05) the anti-inflammatory cytokine IL-10 in the lungs. Song et al. [11] evaluated feeding 1–8% SDP to pregnant mice to attenuate inflammation associated with reproductive performance. Pregnant mice were subjected to transport stress then fed diets with or without SDP during gestation. The transport stress resulted in inflammation as indicated by increased (*p* < 0.05) uterine pro-inflammatory cytokine concentrations of TNFα and IFNγ and reduced (*p* < 0.05) TGF-β1. Feeding SDP to the stressed pregnant mice significantly (*p* < 0.05) reduced TNFα and IFNγ and increased (*p* < 0.05) TGF-β1 resulting in improved pregnancy rate of 40% vs. 15% in control mice. Thus, the results indicate feeding SDP not only impacts the intestinal immune response but, through the common mucosal immune system, modulates inflammation systemically at both the respiratory and reproductive mucosal systems (Table 1).

Altering the microbiota of the intestine may also impact intestinal barrier function, the activation of the gut-associated lymphoid tissue and anti-inflammatory effects. Moretó et al. [27] evaluated whether feeding SDP alters microbial populations using the SEB intestinal mouse model. Supplementation of SDP increased *Lactobacillaceae* and *Lachnospiraceae* (*Firmicutes* phylum). The *Lactobacillaceae* have known probiotic anti-inflammatory effects, while *Lachnospiraceae* have been involved in the regulation of regulatory T lymphocyte homeostasis and the reduction of mucosal permeability [28]. *Porphyromonadaceae* was also increased with SDP [27] and is negatively correlated with intestinal dysfunctions [29]. In contrast, supplementation of SDP decreased *Bifidobacterium* by a 6.6-fold change. The researchers reported that although *Lactobacillus* was increased and *Bifidobacterium* was decreased, the total number of lactic acid-producing bacteria was the same [27]. Tran et al. [30] fed weaned pigs a diet with 5% SDP and reported that fecal microbial communities such as *Lactobaccillus* were increased at d 7, while *Clostridium difficile* at d 28 were decreased (*p* < 0.05) providing information that may partially explain the positive effect associated with feeding SDP on intestinal health and digestibility. Thus, another mechanism of SDP on intestinal inflammation may be through indirectly improving barrier function and modulation of the mucosal immune response from the beneficial effect of SDP on intestinal microbiota. Other research has shown that the gut microbiome can influence intestinal immune responses [31,32] and energy metabolism [33].

In summary, SDP could be improving intestinal health through multiple modes of action including either directly influencing the immune inflammatory response and immunomodulation both locally or systemically and/or through indirectly modifying microbial populations. By reducing intestinal inflammation and improving intestinal microbiota profile, SDP may be directly preventing translocation of bacteria or other antigens, modulating the common mucosal immune system to reduce immune activation both locally and systemically, and/or increasing the presence of bacterial populations that may shift intestinal immune response toward anti-inflammatory.

Regardless of the mechanism, SDP supports anti-inflammatory effects, which result in improved intestinal health as indicated by reduced permeability, increased nutrient transport and systemic immunomodulation to reduce inflammation that leads to improved animal performance.

Spray-dried plasma is a complex mixture of many bioactive proteins and peptides [5,34]. Extracts of bovine colostrum or bovine colostrum whey have been reported to support the innate immune function both in vitro and in vivo [35,36]. The bioactive components of milk originate from blood; thus, proteins such as immunoglobulin, glycoproteins, antimicrobial peptides, growth factors, and other components reported in whey or colostrum could also be contained in SDP. Research with orally fed antimicrobial peptides [37] and protein hydrolysates [38] has reported beneficial effects on gut microbiota, intestinal morphology, animal performance and gut health. Thus, it has not been determined which component in SDP elicits immunomodulation and reduced inflammation to improve well-being of the animal; however, the diverse mixture of bioactive proteins or peptides are likely contributing to these beneficial effects.

## 3. Spray-Dried Plasma and Poultry Production

Immune activation or depression caused by stressors such as deficient housing and/or management, environmental heat or cold stress, disease challenge, mycotoxins, antinutritional factors, transport, or high stocking density can adversely affect economically important production traits such as growth, feed efficiency, mortality, carcass yield, and meat quality in poultry production. A compromised intestinal barrier also results in immune system activation, resulting in reduced intestinal function (i.e., nutrient absorption), which ultimately impacts productive parameters. Depending on the degree of immune activation and/or stress, animals may experience reduced growth [15,16,17], loss of feed efficiency and increased mortality. Thus, utilizing a functional ingredient such as SDP that can support maintenance of intestinal barrier function and improve immune efficiency through various stress conditions would be useful to improve overall performance and productivity.

The following sections review research reporting the use of SDP in feed for poultry housed under both challenging and typical production conditions with many of the studies reporting beneficial effects on intestinal health, performance, and survival. Although, challenge conditions likely varied in severity by study, treatments were randomized across pens to account for variation of challenge within facilities.

### 3.1. Use of Spray-Dried Plasma in Challenge and Stress Conditions

In a study by Campbell et al. [39], broilers were fed SDP only during the first 14 d of age or continuous to market. A natural necrotic enteritis (NE) challenge occurred during the study. The NE challenge was severe, resulting in 56% survival in the control group and 90% in the SDP groups. Feeding SDP only for the first 14 d of life or continuous to market weight during a natural NE challenge resulted in improved (*p* < 0.05) survival compared to control broilers. Feeding SDP improved (*p* < 0.0001) gain and feed efficiency compared to control diets. However, continuous feeding improved (*p* < 0.05) gain and feed efficiency by a higher magnitude compared to 14 d feeding in these severe challenge conditions. The data suggests that the early consumption of SDP resulted in a broiler more resistant to the deleterious effects of high NE challenge. This may be due to improved intestinal health developed during the first days of life and throughout the period.

Beski et al. [40] evaluated the effect of SDP on performance and some physiological and immunological responses during *Salmonella sofia* challenge in broilers. As noted by the researchers, the rearing environment greatly impacts performance and feed efficiency. The researchers fed SDP during the first 14 d of life at either 1 or 2% SDP formulated into the diets. One control group was unchallenged, while all other treatments of control, antibiotic (salinomycin 0.05% + zinc bacitracin 0.033%) and SDP were challenged by oral inoculation with *S. sofia* on d 8, 10, and 12. Broilers fed SDP or antibiotics had a higher body weight (BW) than the challenged control group in the starter and grower phases. At market weight (35 d of age), numerically higher BW and improved feed efficiency was noted for both SDP treatments and antibiotics compared to the challenge control group, and the 2% SDP or antibiotics performed like the unchallenged control. No differences were noted in the immune-associated organ weights of liver, spleen, and thymus, while bursa weight was greater (*p* < 0.05) in broilers fed SDP. On d 13, IgG concentrations tended to be lower (*p* < 0.08) in challenged broilers that consumed 1% SDP. At d 13 and 21, the challenged birds consuming SDP or antibiotics had lower serum IgG concentrations than control groups. Based on the immune measurements, organ weights, and performance measurements, the researchers concluded that SDP fed during the starter period impacted the overall inflammatory response and reduced the overstimulation of the immune system, thus positively impacting broiler performance, the development of the immune organs, and gut health during exposure to high challenge conditions.

Campbell et al. [41] evaluated feeding spray-dried serum (a fraction of SDP) to turkey poults during a systemic immune challenge with *Pasteurella multocida*. The turkey poults challenged on d 35 of age were fed spray-dried serum via the water from d 1 through the challenge period of d 35 to 49. Feeding spray-dried serum via the water improved (*p* < 0.05) survival of challenged poults (94% survival) compared to challenged poults consuming untreated water (63% survival).

Cherian et al. [42] evaluated SDP in commercial farms with a previous history of inclusion body hepatitis (IBH). Spray-dried plasma was included in the starter diet for the first 10 d at 2% and compared to a control starter diet. A common dietary program was fed in the grower and finisher phases. Two experiments were conducted to evaluate mortality, gain, and feed conversion. In both experiments, mortality was numerically reduced on average from 6.5 to 3.9% and average daily gain numerically improved from 52.6 to 56.2 g/d when fed SDP compared to control diet. Feed conversion was evaluated on one site, indicating a 7-point improvement (1.72 vs. 1.79) in efficiency when fed SDP. The researchers concluded that farms with a history of IBH had lower mortality, better gain, and improved feed conversion when SDP was used in the starter diet.

Thus, in a variety of challenge conditions (Table 2), feeding of SDP during the starter feed period improved performance and efficiency and reduced mortality, suggesting that the effects of SDP are likely mediated, at least partially, by improvements in both local and systemic immune responses and by the increased efficiency of nutrient absorption and/or utilization.

### 3.2. Use of Spray-Dried Plasma in Production Conditions

Dietary SDP helps to support the immune response and improve performance with greater benefits noted in animals under challenge conditions [12,13]. Evaluations of SDP in feed for broilers under normal production conditions have also been performed.

Initial SDP titration research was completed by Bregendahl et al. [43] in two floor-pen experiments with 50% clean shaving mixed with 50% soiled litter from on-site turkey production for the first experiment to simulate normal production and use of soiled litter from the first experiment for the second experiment. Levels of SDP consisted of 0, 0.5, 1.0, 1.5, and 2.0% in the starter phase. These levels of SDP were reduced by half in the grower phase and again in the finisher phase. In experiment 1, SDP did not affect growth or carcass measurements. However, in experiment 2, which had higher stress as indicated by the controls having a higher mortality and lower growth than experiment 1, SDP improved (*p* < 0.05) growth rate, feed conversion, flock uniformity, and breast-meat yield. Henn et al. [44] evaluated SDP in two experiments consisting of titrated levels of SDP from 0, 1.5, and 3% SDP in the starter followed by 0 or 0.5% SDP in the grower phase in experiment 1, while experiment 2 had 4 treatments consisting of SDP levels of 0, 0, 0; 1.5, 0, 0; 1.5, 0.5, 0; and 1.5, 0.5, 0.25%, respectively, for the starter, grower and finisher phases. Feeding SDP in Experiment 1 resulted in lower (*p* < 0.05) feed intake and improved (*p* < 0.05) feed conversion from d 1 to 21. Experiment 2 resulted in higher (*p* < 0.05) weight gain from d 1 to 42 for broilers consuming SDP compared to control. They concluded that the addition of SDP can impact the performance of broilers in challenge conditions, with the positive effect mainly in the first stages of life.

Jamroz et al. [45] fed 2 and 4% SDP for 28 d resulting in numerically lower BW compared to control diet. Continued work by Jamroz et al. [46], feeding 2 and 4% SDP in two broiler studies for either 28 or 30 d, respectively, was conducted housing chicks in battery cages. In the first experiment, 4% SDP provided the greatest (*p* < 0.05) BW at d 14. At d 28 or 30, BW was improved (*p* < 0.05) in both experiments. Feed conversion was improved (*p* < 0.05) in experiment 1 when feeding both 2 and 4% SDP compared to control at d 14, while no differences were noted in experiment 1 at d 28 or experiment 2 at d 30. Since the broilers were housed in battery cages, the production stress may have been lower, resulting in smaller treatment differences as reported in previous research by Campbell et al. [13]—who found that battery cage housing resulted in smaller performance differences in response to SDP compared to floor-pen housing.

Beski et al. [47] evaluated the level of feeding SDP to replace a meat meal focusing only on the early broiler starter diets and housing in cages. For the first 10 d, 0, 0.5, 1.0, or 2.0% SDP was included in broiler starter diets with two types of grains (wheat or corn based) and then common diets were fed to market weight at d 35. In the first 10 d and throughout the study to d 35, increasing levels of SDP increased body weight regardless of grains used in the diet. Feed per gain was also improved (*p* < 0.05) from d 10 and 35 due to prior inclusion of SDP in the starter feed. In addition to growth, they reported that broilers fed SDP had longer villi, greater crypt depth and lower villi to crypt ratio than control birds at 24 d regardless of grain-based diet. The authors indicated that feeding SDP stimulated the development of the small intestinal mucosa which lead to better absorption and utilization of nutrients for biological activities, including growth. They concluded that supplementation of starter diets with SDP would benefit the long-term growth of broilers.

Follow up research by Beski et al. [48] evaluated SDP level and duration of feeding in broilers housed in cages. In addition to performance, they determined the effect of SDP on digestive enzymes, digestibility, and intestinal mucosal development. Inclusion levels of 0, 1, or 2% SDP fed for either 5 or 10 d in the starter diet were utilized. In the first 10 d, there was an interaction between level and feeding duration, resulting in improved BW in broilers receiving the 2% SDP for 10 d compared to the other groups. Relative to duration, they reported that feeding SDP for 10 d improved (*p* < 0.01) feed conversion compared to feeding SDP for 5 d. At d 35, birds fed SDP had higher (*p* < 0.06) BW than controls. Digestive enzyme activities and nutrient digestibility were not affected by SDP in this study. The researchers concluded that level and feeding duration improved feed conversion in birds fed SDP for the 10-d period.

Campbell et al. [49] evaluated 0, 0.75, and 1.5% SDP for 14 d in broilers housed in floor pens. The research was focused to only evaluate the starter period, with the study ending on d 14. Increasing levels of SDP up to 1.5% in the starter feed to d 14 positively increased (*p* < 0.05) early broiler growth performance. Average BW at d 14 was 0.33, 0.35, and 0.37 kg for 0, 0.75, and 1.5% SDP, respectively. Most of the improvement (*p* < 0.05) in feed conversion was noted at 0.75% SDP with 1.31, 1.28, and 1.28 for 0, 0.75, and 1.5% SDP, respectively. Walters et al. [50] fed 0. 0.5, 1, 1.5, or 2% SDP for 10 d in floor pens with used litter and fed common diets thereafter to market weight at d 42. Increasing levels of SDP during the starter period increased BW compared to control diet at d 10. The improvement in BW continued through d 28 and 42 with the supplementation of SDP at 1 and 2% yielding 63 and 57 g improvements, respectively. The weight-adjusted feed conversion was improved with 1 and 2% SDP compared to controls.

González-Esquerra et al. [51,52] evaluated duration and SDP level in floor pens housed in a commercial barn. Two trials were conducted, one with normal production challenge and the other during a severe health challenge. The feeding dose of SDP ranged from 1.5 to 6 g/bird being achieved by ranging feeding durations from d 4 to 10 and level from 1 to 3.6% SDP. During normal production challenges, body weight gain (3269 vs. 3143 g) and feed conversion ratio (1.69 vs. 1.75) at d 42 was improved when feeding SDP vs. the control group, respectively. When the broilers experienced a severe health challenge in production, mortality was reduced (*p* < 0.05) from 84% for control to 14% when feeding 6 g of SDP for 10 d. Body weight gain (1.97 vs. 1.26 kg) and feed conversion (1.66 vs. 1.77) were also improved when fed 6 g SDP for 10 d compared to control-fed broilers, respectively. Thus, the data indicates, as in previous research, that the severity of environmental challenge magnifies the beneficial effects of feeding SDP.

Feeding SDP levels over a range of feeding durations in the starter diet resulted in improved BW and feed conversion and reduced mortality. Supplementing SDP in the starter phase is beneficial to improving overall broiler performance. Because various diseases and stress challenges may influence the immune system, intestinal health, and immune efficiency differently, the response of birds to feeding SDP may vary in magnitude. The previous reports show that various levels and duration of feeding SDP have been evaluated in low and high stress production environments and that the optimal SDP dietary level and feeding duration to be used seems to be somewhat dependent on the severity of those challenges.

## 4. Spray-Dried Plasma and Growth-Promoting Antibiotics in Poultry

The use of growth-promoting antibiotics in animal agriculture is being decreased or discontinued due to the concern of generating antimicrobial resistance that could be transferred to humans. Research is actively being conducted to identify alternatives or tools to manage these scenarios. Due to the SDP mode of action that improves intestinal health and supports the immune system, it has been studied as an alternative or as a complement to growth-promoting antibiotics. Several studies have been conducted in pigs demonstrating the beneficial effects of SDP in the presence or absence of growth-promoting antibiotics in feed [8,12,53,54]. Most studies have not reported interactions, suggesting that the effect of SDP is additive to growth-promoting antibiotics.

An evaluation of SDP in broilers in the presence or absence of bacitracin methylene disalicylate (BMD) was conducted by Walters et al. [55] to evaluate the potential use of SDP as an alternative to feed antibiotics or its use in combination with BMD. The study design was a 2 x 2 factorial arrangement of SDP fed at 0 or 2% for 10 d and BMD at 0 or 50 g/ton during the entire study. No interactions were noted between SDP and BMD. The addition of BMD increased (*p* < 0.05) BW at d 10, 28, and 41 and improved (*p* < 0.05) feed conversion during the finishing phase and cumulatively throughout the entire trial. The inclusion of SDP at 2% for the first 10 d increased BW by 18 g and continued to improve BW through d 28 and 41. Body weight uniformity was also improved (*p* < 0.05) with SDP on d 41. Feed conversion was improved at d 10 and cumulative from d 1 to 41 compared to control-fed broilers. The researchers concluded that the beneficial effects of SDP inclusion in the starter diet were noted in BW and feed conversion in diets with or without BMD by the end of the study.

## 5. Conclusions

Spray-dried plasma is a unique functional ingredient that supports and maintains intestinal health and functionality by mechanisms that modulate the local and systemic immune system. The reviewed literature discussed the possible modes of action of SDP and its effects on productive measures in both challenged and non-challenged birds as affected by supplemental SDP levels and feeding duration. Collectively, the current literature suggests that SDP improves early intestinal health and supports an efficient immune system response locally at the intestine and systemically, therefore reducing the need for nutrients to be diverted for use to support the activation of the immune system and diverting them towards productive functions, thereby improving overall performance and the well-being of poultry.

## Figures and Tables

**Table 1:** Summary of spray-dried plasma mode of action.

Reference	Challenge/Stress	Specie	Site	Spray-Dried Plasma Action
Pérez-Bosque et al., 2004 [19]	*Staphylococcus aureus* enterotoxin B (SEB)	Rats	Intestine	↓ fecal water content ↓ activated T helper cells
Garriga et al., 2005 [21]	SEB	Rats	Intestine	↑ glucose absorption
Pérez-Bosque et al., 2006 [9]	SEB	Rats	Intestine	↓ intestinal permeability ↑ tight junction protein expression
Pérez-Bosque et al., 2010 [23]	SEB	Rats	Intestine	↑ IL-10, TGF-β1 ↓ TNFα, IL-6, LTB4
Pérez-Bosque et al., 2016 [24]	SEB	Mice	Intestine	↑ IL-10, TGF-β1, Foxp3, NF-κB phosphorylation ↓ TNFα, MAdCAM-1, ICAM-1
Pérez-Bosque et al., 2010 [25]	SEB	Rats	Intestine	↑ β-defensin 1, cryptdin 4
Maijó et al., 2012 [10]	Lipopolysaccharide (LPS)	Mice	Lung	↓ TNFα, IL-6, IL-1α, chemokines (CCL2, CCL3, and CCL4)
Maijó et al., 2012 [26]	LPS	Mice	Lung	↑ IL-10, TGF-β1
Song et al., 2015 [11]	Transport Stress	Mice	Uterus	↑ TGF-β1, pregnancy rate ↓ TNFα, IFNγ

IL-10, Interleukin 10; TGF- β1, transforming growth factor β1; TNFα, tumor necrosis factor α; IL-6, Interleukin 6; LTB4, leukotriene B4; Foxp3, forkhead box P3; NF-κB, nuclear factor kappa B; MAdCAM-1, mucosal addressin cell adhesion molecule 1; ICAM-1, intercellular adhesion molecule 1; IL-1α, Interleukin 1α; CCL, chemokine ligands; IFNγ, interferon γ.

**Table 2:** Summary of spray-dried plasma effects during pathogen challenge in poultry.

Reference	Pathogen Challenge	Spray-Dried Plasma Effects
Campbell et al., 2006 [39]	Necrotic Enteritis	↑ survival
Beski et al., 2016 [40]	*Salmonella*	↑ body weight gain, ↓ feed conversion ratio
Campbell et al., 2004 [41]	*Pasteurella multocida*	↑ survival
Cherian et al., 2019 [42]	Inclusion body hepatitis	↓ mortality, feed conversion ratio, ↑ body weight gain
